# Highly efficient and stable organic light-emitting diodes with a greatly reduced amount of phosphorescent emitter

**DOI:** 10.1038/srep09855

**Published:** 2015-05-18

**Authors:** Hirohiko Fukagawa, Takahisa Shimizu, Taisuke Kamada, Shota Yui, Munehiro Hasegawa, Katsuyuki Morii, Toshihiro Yamamoto

**Affiliations:** 1 Japan Broadcasting Corporation (NHK), Science & Technology Research Laboratories, 1-10-11 Kinuta, Setagaya-ku, Tokyo 157-8510, Japan; 2 Tokyo University of Science, 1–3 Kagurazaka, Tokyo 162-8610, Japan; 3 Nippon Shokubai Co., Ltd., 5-8 Nishi Otabi-cho, Suita, Osaka, 564-8512, Japan

## Abstract

Organic light-emitting diodes (OLEDs) have been intensively studied as a key technology for next-generation displays and lighting. The efficiency of OLEDs has improved markedly in the last 15 years by employing phosphorescent emitters. However, there are two main issues in the practical application of phosphorescent OLEDs (PHOLEDs): the relatively short operational lifetime and the relatively high cost owing to the costly emitter with a concentration of about 10% in the emitting layer. Here, we report on our success in resolving these issues by the utilization of thermally activated delayed fluorescent materials, which have been developed in the past few years, as the host material for the phosphorescent emitter. Our newly developed PHOLED employing only 1 wt% phosphorescent emitter exhibits an external quantum efficiency of over 20% and a long operational lifetime of about 20 times that of an OLED consisting of a conventional host material and 1 wt% phosphorescent emitter.

The efficiency of organic light-emitting diodes (OLEDs) has been under intense investigation with the aim of reducing the power consumption of OLED-related devices such as displays and lighting systems. The first OLED, reported over 20 years ago, consisted of conventional fluorescent materials^1^. Although OLEDs fabricated using conventional fluorescent materials now have sufficient reliability for practical use, their internal electroluminescence quantum efficiency (*η*_int_), defined as the number of photons generated per injected carrier, is limited to 25% because of the exciton branching ratio of singlet excited states under electrical excitation^2^. On the other hand, *η*_int_ of 100% is expected by employing phosphorescent materials with heavy atoms and thermally activated delayed fluorescence (TADF) materials^3^,^4^,^5^. In 1999, an efficient phosphorescent OLED (PHOLED) using iridium complexes was first demonstrated, and *η*_int_ of almost 100% was achieved in the following year^6^,^7^. Nowadays, PHOLEDs with *η*_int_ of about 100% can easily be fabricated with a device architecture that can confine charges and excitons inside the phosphorescence-emitting layer; however, reports on PHOLEDs with high operational stability have been limited^8^,^9^. Another issue in the practical application of PHOLEDs is that the cost of the phosphorescent emitter tends to be high since the crustal abundance of heavy atoms used in phosphorescent emitters is low. In recent years, on the other hand, *η*_int_ of almost 100% has been achieved by employing TADF materials, which are synthesized without the use of heavy atoms^10^,^11^,^12^. TADF materials harness both singlet and triplet excitons to induce light emission through fluorescence decay channels since the energy gap between singlet and triplet excited states (Δ*E*_ST_) is minimized^5^,^10^,^11^. Two main methods have been proposed for minimizing Δ*E*_ST_: one is the use of a single molecule consisting of donor and acceptor units^5^,^10^,^11^, and the other is exciplex formation between electron-donating and electron-accepting molecules^13^. Although several OLEDs with *η*_int_ of almost 100% have been achieved by employing TADF materials, there have been few reports on highly operational stable OLEDs, as is the case with PHOLEDs.

Recently, highly efficient OLEDs with long operational stability have been demonstrated, which utilize materials with a small Δ*E*_ST_. The efficiency and lifetime of fluorescent OLEDs have been reported to be improved by employing a TADF material as an assist dopant^14^. Also, a highly efficient and stable PHOLED with low-voltage operation has been demonstrated by employing an exciplex with a small Δ*E*_ST_ as the host^15^. Although such highly efficient and stable OLEDs have been demonstrated, the emitting layer is composed of at least three materials, which makes both the composition of the emitting layer and the fabrication process more complicated^16^,^17^,^18^. On the other hand, it has been reported that a single TADF material, which consists of donor and acceptor units, can also be used for the host material in a PHOLED^19^,^20^,^21^,^22^. The main advantage of such a PHOLED has been proposed to be the lower driving voltage than that of a PHOLED using a conventional host, which is due to the relatively small band gap of the TADF material as compared with that of a conventional host with similar triplet energy^19^,^20^,^21^. We have recently reported that a highly efficient and stable PHOLED can be realized using a TADF material as the host since the electrically excited triplet excitons of the host, which are key elements in operational degradation^23^, are transferred rapidly to the dopant following the Förster process via reverse intersystem crossing (RISC) from the triplet to singlet states (Fig. 1a)^24^. If the Förster energy transfer from the excited states of the host to the phosphorescent dopant can be utilized effectively, there is a strong possibility of reducing the concentration of the emitter dopant, which is 6–15 wt% in conventional PHOLEDs (Fig. 1c)^6^,^7^,^8^,^9^,^25^,^26^, to 1–3 wt%, similar to that in fluorescent OLEDs^27^,^28^,^29^. This will significantly decrease the amount of heavy atoms used, resulting in a lower cost. Moreover, the configuration of the emitting layer in this PHOLED is relatively simple since the emitting layer is composed of only a TADF material and a small amount of phosphorescent emitter (Fig. 1b).

On the basis of the energy transfer between the TADF material and the phosphorescent emitter, we demonstrate highly efficient and operationally stable PHOLEDs with only 1 wt% phosphorescent emitter. A highly efficient green PHOLED exhibiting an operational lifetime of over 10,000 h with an initial luminance of 1,000 cd m^−2^ is realized using 2-biphenyl-4,6-bis(12-phenylindolo[2,3-a]carbazol-11-yl)-1,3,5-triazine (PIC-TRZ) as the host and 1 wt% tris[4-(*o*-tolyl)pyridine]iridium(III) [Ir(mppy)_3_] as the emitter^5^. This operational lifetime is about 20 times longer than that for a 1-wt%-doped PHOLED using the conventional host material 4,4′-bis(9-carbazolyl)-2,2′-biphenyl (CBP). The TADF host/phosphorescent emitter configuration is found to be more suitable for practical use since the demonstrated PHOLED performances are independent of the emitter concentration from 1 to 6 wt%, eliminating the need for a strictly controlled fabrication process. The generality of this emitting layer configuration is also confirmed by demonstrating an efficient and stable red PHOLED employing PIC-TRZ as the host and a small amount of a red phosphorescent emitter based on platinum.

## Results

### Host-dependent characteristics of green PHOLEDs

**Figure 2:**
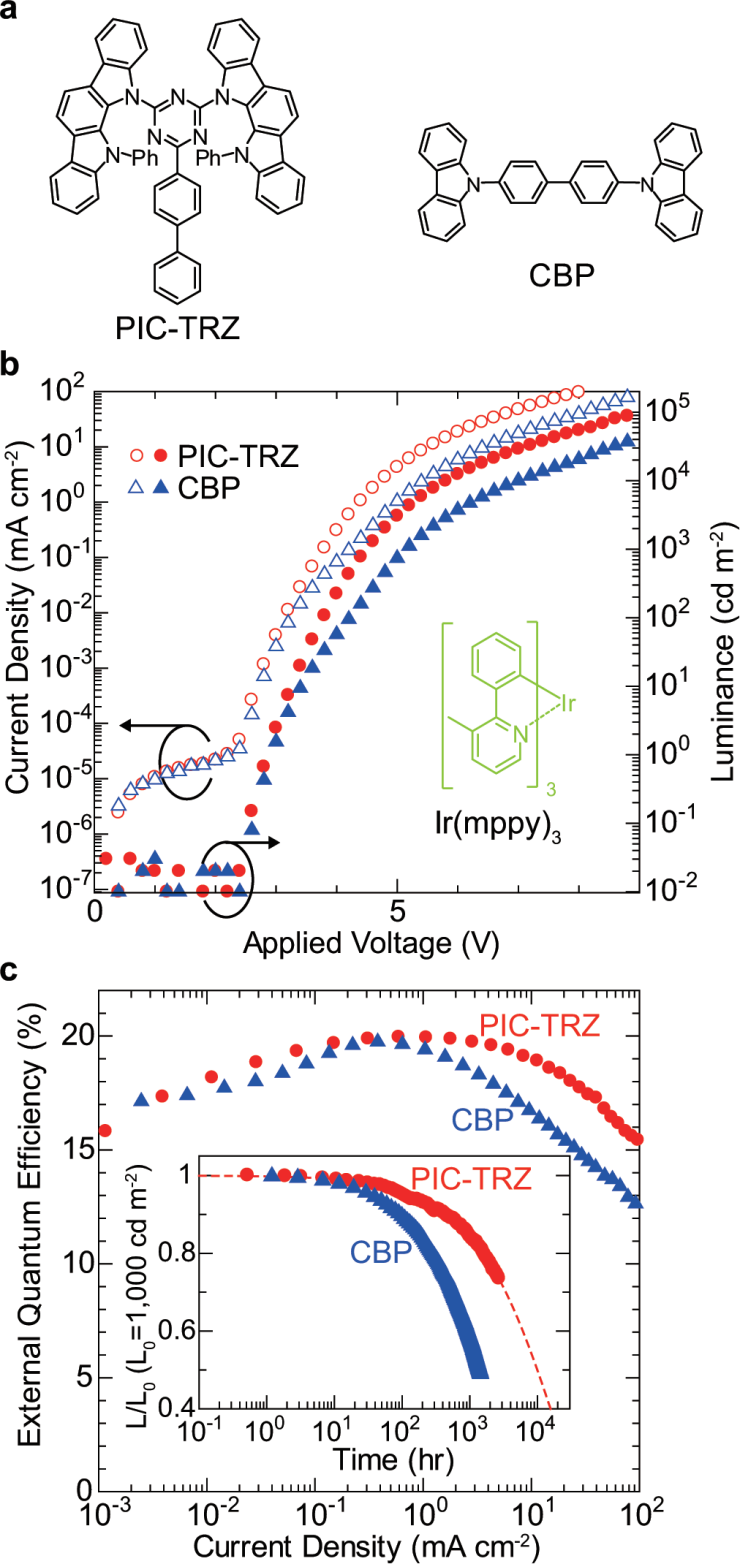
Performance of PHOLEDs with different hosts. **(a)** Molecular structures of host material used in this study. **(b)** Current density (left, open symbols) and luminance (right, filled symbols)–voltage characteristics of PHOLEDs. **(c)** External quantum efficiency–current density curves of PHOLEDs. Inset: Luminance–time characteristics for devices under a constant dc current with an initial luminance of 1,000 cd m^−2^.

The device characteristics of green PHOLEDs using two hosts are compared, as shown in Fig. 2a. First, we fabricated 6-wt%-Ir(mppy)_3_-doped PHOLEDs using PIC-TRZ as the TADF material and the conventional host CBP to discuss the effect of RISC on the device characteristics^5^. CBP is suitable as a comparable host since not only do CBP and PIC-TRZ have a carbazole unit in their molecular structures, but also the triplet energy of CBP and PIC-TRZ is almost the same^24^. The OLEDs were composed of multiple layers of indium tin oxide (ITO), Clevios HIL 1.5 (supplied by Heraeus Holding GmbH, 30 nm), 4,4′-bis[*N*-(1-naphthyl)-*N*-phenylamino]-biphenyl (α-NPD, 20 nm), HTEB-2 (supplied by Kanto Chemical Co., Inc., 10 nm), 6 wt% Ir(mppy)_3_:host (25 nm), 1,3,5-tris(*N*-phenylbenzimidazol-2-yl)benzene (TPBi, 35 nm), LiF (0.8 nm), and Al (100 nm) (details are shown in Supplementary Fig. S1). The triplet energies of the hosts, namely, PIC-TRZ and CBP, are larger than that of the emitter dopant Ir(mppy)_3_ (details are shown in Supplementary Fig. S2)^5^,^30^. The triplet energies of the carrier-transporting materials HTEB-2 and TPBi are also larger than that of Ir(mppy)_3_^24^. Thus, the triplet energy transfer from Ir(mppy)_3_ to the other materials is completely suppressed and the energy is confined in Ir(mppy)_3_.

Figure 2b shows the current density (*J*)–voltage (*V*)–luminance (*L*) characteristics of the PHOLEDs. The *J*–*V*–*L* characteristics show that the PHOELD using PIC-TRZ has a lower driving voltage than the PHOLED using CBP, as previously reported^19^. The external quantum efficiency (*η_EQE_*) vs *J* characteristics of both PHOLEDs are shown in Fig. 2c. The normalized luminance of the PHOLEDs as a function of operating time with an initial luminance of 1,000 cd m^−2^ is also shown in the inset. Both PHOLEDs exhibit the highest *η*_EQE_ of about 20%, which is equivalent to an internal electroluminescence quantum efficiency of about 67–100%, assuming a light outcoupling efficiency of 20–30%^31^,^32^. In the PHOLED using CBP, efficiency roll-off caused by triplet-triplet annihilation is observed^33^. On the other hand, the PHOLED using PIC-TRZ exhibits a smaller roll-off, which may be attributed to the rapid energy transfer from PIC-TRZ to Ir(mppy)_3_ and/or the wider charge recombination zone resulting from the bipolar features of PIC-TRZ^19^,^24^. The operational lifetime LT50, which is defined as the time for the luminance to decay to 50% of the initial luminance, strongly depends on the host material. The LT50 value was only 1,500 h for the PHOLED using CBP. In contrast, LT50 for the PHOLED using PIC-TRZ was estimated using the well-known stretched exponential decay function to be over 10,000 h^34^, about seven times longer than that for the PHOLED using CBP. In the PHOLED using PIC-TRZ, the electrically excited host triplets, which are key elements in operational degradation, are transferred rapidly to Ir(mppy)_3_ following the Förster process, resulting in high operational stability^24^.

### Dopant-concentration-dependent characteristics

If the electrically excited host triplets in the PHOLED are transferred to the emitting dopant following the Förster process, the emitting dopant concentration can be reduced to the same amount as in conventional fluorescent OLEDs^27^,^28^,^29^. To verify this assumption, we evaluated the dopant-concentration-dependent PHOLED characteristics using PIC-TRZ and CBP as hosts. The key data for these PHOLEDs are listed in Table 1. The PHOLED characteristics using CBP clearly depend on the dopant concentration, as previously reported (details are shown in Supplementary Fig. S3)^25^. The driving voltage at a luminance of 1,000 cd m^−2^ decreases with increasing dopant concentration, which may be caused by the carrier trapping and/or direct carrier recombination by the dopant^25^,^35^. In the PHOLED using PIC-TRZ, on the other hand, not only the driving voltage but also *η_EQE_* is almost independent of the dopant concentration from 1 to 6 wt% (details are shown in Supplementary Fig. S4). Thus, we speculate that the energy transfer from the host to the dopant dominates the emission in the PHOLED using PIC-TRZ rather than direct carrier recombination by the dopant. The *η_EQE_* of the 10-wt%-doped PHOLED using PIC-TRZ is relatively lower than that of the other PHOLEDs, which may be caused by triplet-triplet annihilation^33^. In light of the operational stability, LT50 of the PHOLED using CBP clearly depends on the dopant concentration. The 6-wt%-doped PHOLED exhibits the longest operational stability, and the LT50 of the 1-wt%-doped PHOLED is much shorter. In the case of the PHOLED using PIC-TRZ, on the other hand, the operational stability is also almost independent of the dopant concentration from 1 to 6 wt%. In particular, the LT50 of over 10,000 h is expected even for the 1-wt%-doped PHOLED, which is about 20 times longer than that of the 1-wt%-doped PHOLED using CBP. The lower limit of the dopant concentration is expected to be about 1 wt% from the fact that the efficiency of a 0.5-wt%-doped PHOLED using 2-phenyl-4,6-bis(12-phenylindolo[2,3-a] carbazole-11-yl)-1,3,5-triazine (PBICT)^20^, which is a similar TADF material to PIC-TRZ, is significantly lower than that of a 1-wt%-doped PHOLED using PBICT (details are shown in Supplementary Fig. S5). The stable operation of the PHOLED is realized with a small amount of dopant by utilizing the efficient energy transfer from the triplet-excited states of PIC-TRZ to the dopant via the singlet-excited state of PIC-TRZ. Thus, we demonstrated the feasibility of a highly efficient and operationally stable green PHOLED with a greatly reduced amount of phosphorescent dopant by employing a TADF material as the host. Moreover, it can be seen from Table 1 and Supplementary Fig. S4 that high performances are obtained without strictly controlling the dopant concentration. Thus, it is reasonable to conclude that the proposed emitting layer configuration is suitable for practical use.

### Transient photoluminescence analysis

**Figure 3:**
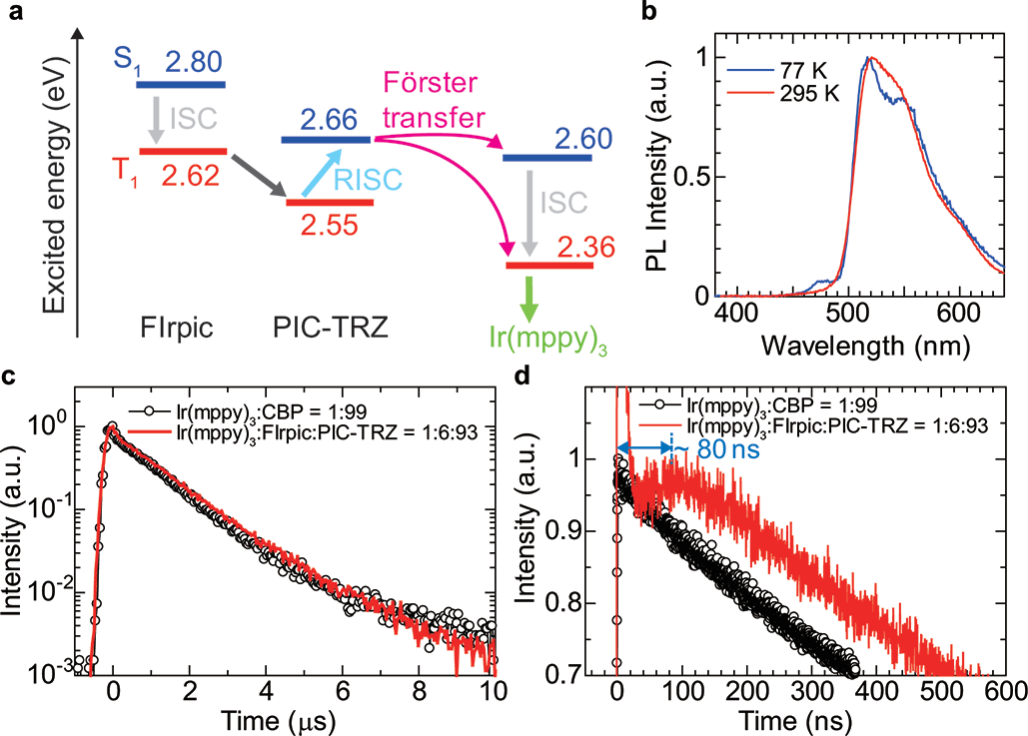
Analysis of energy transfer. **(a)** Energy diagram of singlet excited states (S_1_), triplet excited states (T_1_), and energy transfer process in three-in-one film. **(b)** Temperature dependence of PL spectrum of three-in-one film, which has the structure 6 wt%-doped FIrpic: 1 wt%-doped Ir(mppy)_3_: PIC-TRZ film. **(c, d)** Transient PL curve of the three-in-one film. The emission was detected at λ = 600 nm to eliminate the effect of fluorescence from PIC-TRZ.

The energy transfer rate from the excited states of PIC-TRZ to the small amount of Ir(mppy)_3_ is also verified using an analysis method that we previously reported^24^. We measured the photoluminescence (PL) decay of a three-in-one film with the structure 6-wt%-doped iridium (III)bis [(4,6-di-fluoropheny)-pyridinato-*N*,C2′]picolinate (FIrpic)^36^: 1-wt%-doped Ir(mppy)_3_: PIC-TRZ. As we have already reported, the energy transfer mechanism of triplet excitons between PIC-TRZ and Ir(mppy)_3_ can be clarified by PL decay measurement of the three-in-one film. It is likely that FIrpic triplets are transferred following the Förster process via PIC-TRZ singlet states, as shown in Fig. 3a, since no FIrpic emission is observed in the PL spectrum (Fig. 3b). We also investigated the temperature dependence of the PL spectrum of the three-in-one film to discuss the effect of the RISC in the host on the energy transfer as shown in Fig. 3b (details are shown in Supplementary Fig. S6). The intensity of FIrpic emission at about λ = 470 nm increases with decreasing the temperature, which indicates that the efficiency of energy transfer from PIC-TRZ to Ir(mppy)_3_ is lower at low temperatures. Since the energy difference between FIrpic triplet states and PIC-TRZ triplet states is smaller than Δ*E*_ST_ for PIC-TRZ, both the RISC in PIC-TRZ and the back energy transfer from PIC-TRZ triplet states to FIrpic triplet states are possible, especially at low temperatures^30^. A similar PL spectrum and back energy transfer have been observed in the energy transfer process among FIrpic, CBP and Ir(mppy)_3_, where the efficiency of energy transfer from the CBP triplet states to Ir(mppy)_3_ is low^24^. Thus, the thermally activated RISC in PIC-TRZ plays a key role in the efficient energy transfer from the PIC-TRZ triplet states to Ir(mppy)_3_. The PL decay results are compared with the PL decay for CPB doped with 1 wt% Ir(mppy)_3_, where the CBP singlet excitons are simply transferred by the Förster process. As shown in Fig. 3c, the PL decay for λ = 600 nm over a period of 10 μs is similar, whereas a clear difference is observed in the first 600 ns (Fig. 3d). In the transient PL characteristics of the three-in-one film, the emission intensity first increases for 80 ns and then decreases. The short transient decay observed in the first 20 ns originates from the fluorescence of PIC-TRZ, the lifetime of which is significantly reduced owing to the transfer of energy to Ir(mppy)_3_^37^. Since the ISC in the Ir complex is extremely rapid^38^ and the singlet energy of PIC-TRZ (2.66 eV) is larger than the triplet energy of FIrpic (2.62 eV), this peculiar characteristic is caused by a number of energy transfer processes such as [FIrpic triplet → PIC-TRZ triplet → PIC-TRZ singlet → Ir(mppy)_3_] singlet or Ir(mppy)_3_ triplet. It was demonstrated that most of the PIC-TRZ triplets are transferred to Ir(mppy)_3_ within 80 ns via PIC-TRZ singlets in the PHOLED using PIC-TRZ. Although the Dexter process from PIC-TRZ triplets to Ir(mppy)_3_ is possible in the PHOLED, we conclude that the RISC of PIC-TRZ makes the energy transfer more efficient and faster. In the PHOLED using PIC-TRZ, the energy transfer takes much less time than the triplet-state lifetime of conventional host molecules (μs order). The electrically excited host triplets, which are key elements in operational degradation^23^, are transferred rapidly to the dopant following the Förster process, resulting in the high operational stability of the 1-wt%-doped PHOLED using PIC-TRZ.

### TADF-host-dependent device characteristics

**Figure 4:**
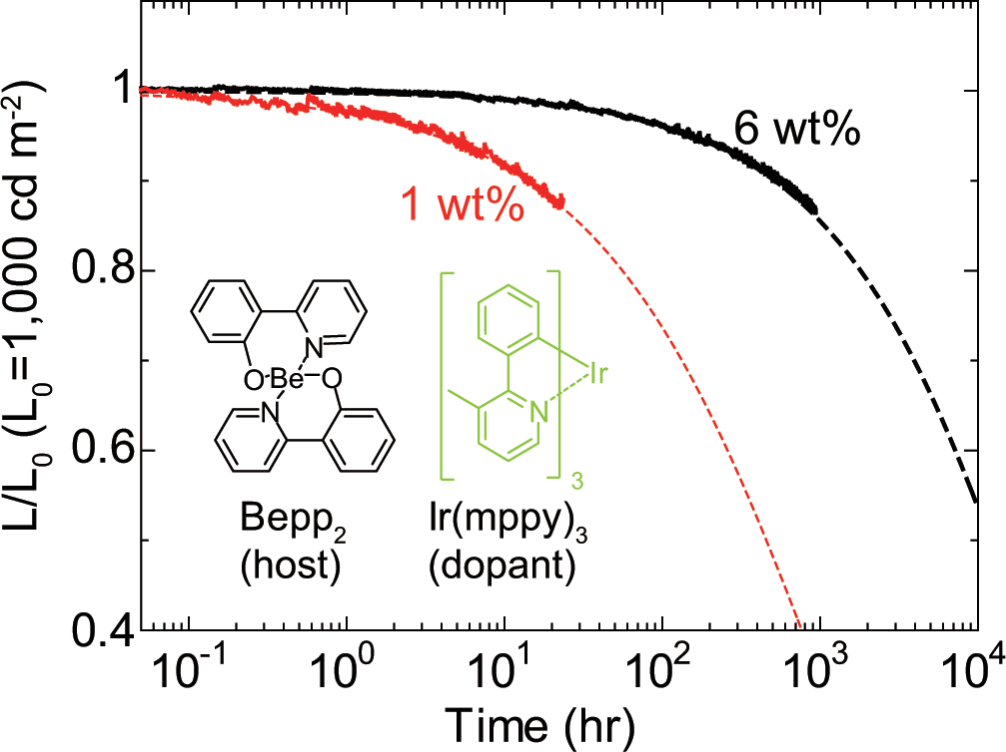
Operational stability of PHOLEDs with metal complex host. Luminance–time characteristics for PHOLED using Bepp_2_ as the host under a constant dc current with an initial luminance of 1,000 cd m^−2^.

Here we discuss the host materials suitable for PHOLEDs with a reduced amount of dopant. There are three main types of TADF materials: molecules with an intermolecular donor-acceptor structure (exciplexes)^13^,^15^,^16^,^17^,^18^, metal complexes^24^,^39^ and molecules with an intramolecular donor-acceptor structure^4^,^5^,^10^,^11^,^12^. By utilizing PIC-TRZ^5^, we demonstrate that TADF materials with an intramolecular donor-acceptor structure can be effective hosts for PHOLEDs with a reduced amount of dopant. In addition, a highly efficient and stable PHOLED employing only 1 wt% emitter has been realized using 5,12-dihydro-12-(4,6-diphenyl-1,3,5-triazin-2-yl)-5-phenylindolo[3,2-a]carbazole (PIC-TRZ2) as the host (details are shown in Supplementary Fig. S7)^40^. In contrast, the other two TADF materials were found to be unsuitable for use as hosts in highly efficient and stable PHOLEDs with a reduced amount of dopant. Figure 4 shows the dopant-concentration-dependent operational stability of PHOLEDs using the metal complex bis[2-(2-hydroxyphenyl)-pyridine]beryllium (Bepp_2_) as the host. The device configuration of the PHOLEDs using Bepp_2_ is the same as that of a previously reported PHOLED^24^. The operational stability of the 1-wt%-doped PHOLED is much shorter than that of the 6-wt%-doped PHOLED. This tendency was observed not only in the PHOLED using Bepp_2_ but also in the PHOLED using other metal complexes^39^. In the case of PHOLEDs using an exciplex as the host, it has already been demonstrated that the efficiency of a PHOLED employing 1 wt% emitter is lower than that of a conventionally doped PHOLED^17^. Thus, we conclude that the only strong candidate host materials suitable for PHOLEDs with a reduced amount of dopant are TADF materials with an intramolecular donor-acceptor structure. A detailed discussion of the difference between PHOLEDs using TADF materials with an intramolecular donor-acceptor structure and PHOLEDs using the other two types of TADF materials will be given elsewhere.

### PHOLED characteristics using red platinum complex

**Figure 5:**
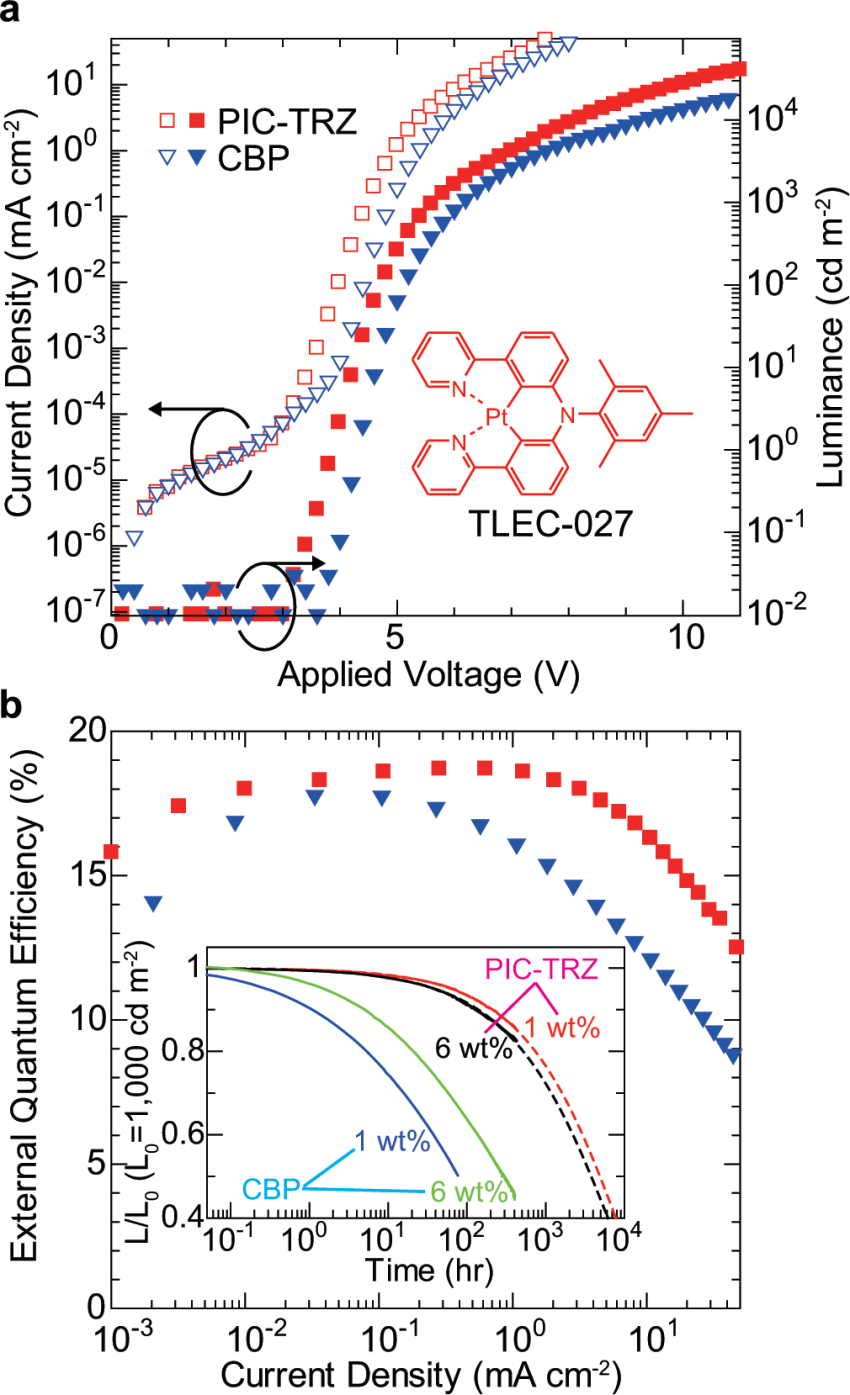
Performance of PHOLEDs with red emitter. **(a)** Current density (left, open symbols) and luminance (right, filled symbols)–voltage characteristics of PHOLEDs. **(b)** External quantum efficiency–current density curves of PHOLEDs. Inset: Luminance–time characteristics for devices under a constant dc current with an initial luminance of 1,000 cd m^−2^.

It is important to demonstrate that a highly stable PHOLED with a reduced amount of emitter can be realized using several kinds of phosphorescent emitter. This is because the performances of PHOLEDs using phosphorescent emitters other than Ir complexes have significantly improved in recent years^41^,^42^. Thus, we evaluated the device characteristics of a PHOLED using another phosphorescent dopant, [[2,4,6-trimethylethylphenylimino]bis[6-(2-pyridinyl-κN)-2,1-phenylene-κC]]platinum(II) (TLEC-027), which is a platinum-based red phosphorescent dopant, to verify the applicability of the TADF host to another phosphorescent emitter^39^. We used PIC-TRZ and CBP as host materials, as in the case of the green PHOLEDs. The device configuration of the red PHOLEDs is the same as that of the green PHOLEDs except for the α-NPD thickness of 30 nm. Figure 5a shows the *J*–*V*–*L* characteristics of the 1-wt%-doped PHOLEDs. The Commission Internationale de l’Eclairage (CIE) coordinates of a PHOLED using PIC-TRZ at 1,000 cd m^−2^ are (0.65, 0.35). As in the case of the green PHOLEDs, the PHOLED using PIC-TRZ exhibits a lower driving voltage than the PHOLED using CBP^19^. The *η_EQE_* vs *J* characteristics of the 1-wt%-doped PHOLEDs are shown in Fig. 5b. The maximum *η_EQE_* of 18.7%, which is a high value for a TLEC-027-based PHOLED, is obtained for the PHOLED using PIC-TRZ^39^. The operational stability of not only 1-wt%-doped PHOLEDs but also 6-wt%-doped PHOLEDs is shown in the inset of Fig. 5b. LT50 for the 1-wt%-doped PHOLED using PIC-TRZ was estimated to be about 5,000 h, which is much longer than that for the PHOLEDs using CBP. In the case of the PHOLEDs using CBP, LT50 for the 1-wt%-doped PHOLED is much shorter than that for the 6-wt%-doped PHOLED. On the other hand, LT50 for the 1-wt%-doped PHOLED using PIC-TRZ is slightly longer than that for the 6-wt%-doped PHOLED. A highly efficient and stable red PHOLED is thus demonstrated by combining the TADF material with a small amount of the platinum-complex emitter. Although relatively high operational stability is obtained, the operational stability of the TLEC-027-based PHOLED is expected to be improved by replacing PIC-TRZ with another more suitable TADF material as the host. More efficient energy transfer from the excited states of the host to those of TLEC-027 is expected by employing another TADF material with a narrower band gap than PIC-TRZ since the Förster energy transfer rate strongly depends on the spectral overlap between the fluorescent spectrum of the host and the absorption spectral features arising from the metal-to-ligand charge-transfer transitions (details are shown in Supplementary Fig. S2)^14^,^43^,^44^. Practicable full-color efficient PHOLEDs will be realized if a suitable TADF material is combined with a phosphorescent dopant.

## Discussion

Our results show that highly efficient and stable PHOLEDs can be realized by combining a TADF material as the host and a small amount of phosphorescent emitter. One of the main aims of developing TADF materials has been to provide an alternative to phosphorescent emitters with heavy atoms, which have rather high costs. Highly efficient OLEDs have recently been realized without the use of heavy atoms by employing a TADF material as emitter^45^, and it has also been found that the amount of the phosphorescent emitter used in the PHOLED can be significantly decreased by employing a TADF material with an intramolecular donor-acceptor structure as the host. Also, there is a possibility that the device characteristics of blue PHOLEDs, the greatest challenge in the practical application of PHOLEDs, can be greatly improved by employing a blue TADF material as the host. The range of TADF materials suitable for use as a phosphorescent host is expected to increase since TADF materials are under intense investigation^45^. Although the efficiency of a fluorescent OLED employing PIC-TRZ as an emitter is not particularly high^5^, PIC-TRZ can act as an efficient host in PHOLEDs. The design strategies for TADF materials suitable for use as a phosphorescent host will be clarified by examining the characteristics of PHOLEDs with several TADF material/phosphorescent dopant configurations.

In summary, the proposed host/dopant configuration is a strong candidate for use in practical OLEDs in the near future. This is because highly efficient and stable OLED can be realized using a simple emitting layer configuration without having to strictly control the dopant concentration while preparing the emitting layer.

## Methods

### Fabrication of OLEDs

The OLEDs were developed on a glass substrate coated with a 100-nm-thick indium tin oxide (ITO) layer having a sheet resistance of 10 ohm/square. Prior to the fabrication of the organic layers, the substrate was cleaned with ultrapurified water and organic solvents, and treated with a UV-ozone ambient. To reduce the possibility of electrical shorts within the device, Clevios HIL 1.5 was spun onto the substrate to form a 30-nm-thick layer.

The other organic layers were sequentially deposited onto the substrate without breaking the vacuum at a pressure of about 10^−5^ Pa. After the organic layers were formed, a 0.8-nm-thick LiF layer and a 100-nm-thick Al layer were deposited as the cathode. The devices were encapsulated using a UV-epoxy resin and a glass cover within a nitrogen atmosphere after cathode formation.

### Device characterization

The EL spectra and luminance were measured with a spectroradiometer (Minolta CS-1000). A digital source meter (Keithley 2400) and a desktop computer were used to operate the devices. We assumed that the emission from the OLED was isotropic, so that the luminance was Lambertian, and we calculated *η*_EQE_ from the luminance, current density, and EL spectra.

### Photoluminescence measurement

The organic films used for optical measurements, which had a thickness of 50 nm, were fabricated on clean quartz substrates by thermal evaporation. The PL spectra of the films were recorded using a spectrofluorometer (Horiba Jobin Yvon, FluoroMax-4). The transient PL characteristics over 10 μs (shown in Fig. 3c) were measured using a streak camera (Hamamatsu, C4334). Parts of the transient PL characteristics (over 600 ns, as shown in Fig. 3d) were measured using the above-mentioned spectrofluorometer. The excitation wavelength of all the PL measurements was 350 or 355 nm.

## Author Contributions

The experiments were convinced and designed by H.F. and T.S., and were carried out by T.K. and S.Y.. M.H., K.M. and T.Y. provided experimental support and suggestions. H.F. and T.S. wrote the manuscript.

## Additional Information

**How to cite this article**: Fukagawa, H. *et al*.Highly efficient and stable organic light-emitting diodes with a greatly reduced amount of phosphorescent emitter. *Sci. Rep.* **5**, 9855; doi: 10.1038/srep09855 (2015).

## Supplementary Material

Supplementary Information Supplementary Figures

## Figures and Tables

**Figure 1:**
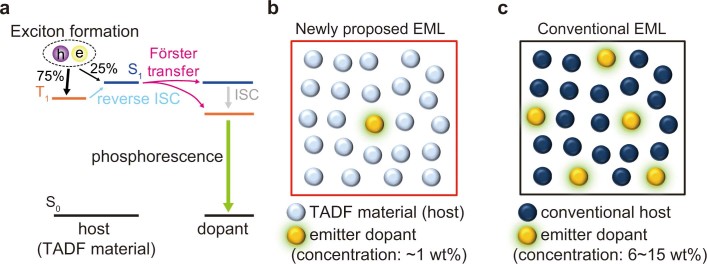
Schematic illustration of energy transfer process and emitting layer (EML). **(a)** Energy transfer process from TADF material to phosphorescent emitter dopant. **(b)** EML in phosphorescent OLED with TADF host. **(c)** EML in phosphorescent OLED with conventional host.

**Table 1:** Performance of PHOLEDs with different dopant concentrations.

		Performance at around 1,000 cd m^−2^					
Host	Dopant concentration (wt%)	Voltage (V)	J (mA cm^−2^)	*η_EQE_* (%)	CE (cd A^−1^)	CIE	LT50 (h)
PIC-TRZ	1	4.5	1.4	20.3	74	(0.34, 0.62)	>10,000*
	3	4.7	1.2	20.0	73	(0.34, 0.62)	>10,000*
	6	4.5	1.4	19.9	72	(0.35, 0.62)	>10,000*
	10	4.5	1.6	17.8	64	(0.36, 0.61)	6,500*
CBP	1	5.6	1.5	19.8	72	(0.32, 0.62)	500
	6	5.2	1.6	19.1	70	(0.34, 0.62)	1,500
	10	5.0	1.4	19.0	68	(0.34, 0.62)	500

CE, current efficiency; CIE, Commission Internationale de I’Eclairage; *, estimated LT50.
