# Supplementary material for: Highly efficient and stable organic light-emitting diodes with a greatly reduced amount of phosphorescent emitter
Source: Sci Rep. 2015 May 18;5:9855. doi: 10.1038/srep09855 (PMC4434910; doi:10.1038/srep09855)
Supplement: Supplementary Information — Supplementary Figures [file srep09855-s1.pdf]

Supplementary Information

Highly efficient and stable organic light-emitting diodes with a greatly reduced amount of phosphorescent emitter

Hirohiko Fukagawa[1], Takahisa Shimizu[1], Taisuke Kamada[2], Shota Yui[2], Munehiro Hasegawa[3], Katsuyuki Morii[3] and Toshihiro Yamamoto[1]

[1] Japan Broadcasting Corporation (NHK), Science & Technology Research Laboratories, 1-10-11 Kinuta, Setagaya-ku, Tokyo 157-8510, Japan

[2] Tokyo University of Science, 1–3 Kagurazaka, Tokyo 162-8610, Japan

[3] Nippon Shokubai Co., Ltd., 5-8 Nishi Otabi-cho, Suita, Osaka, 564-8512, Japan

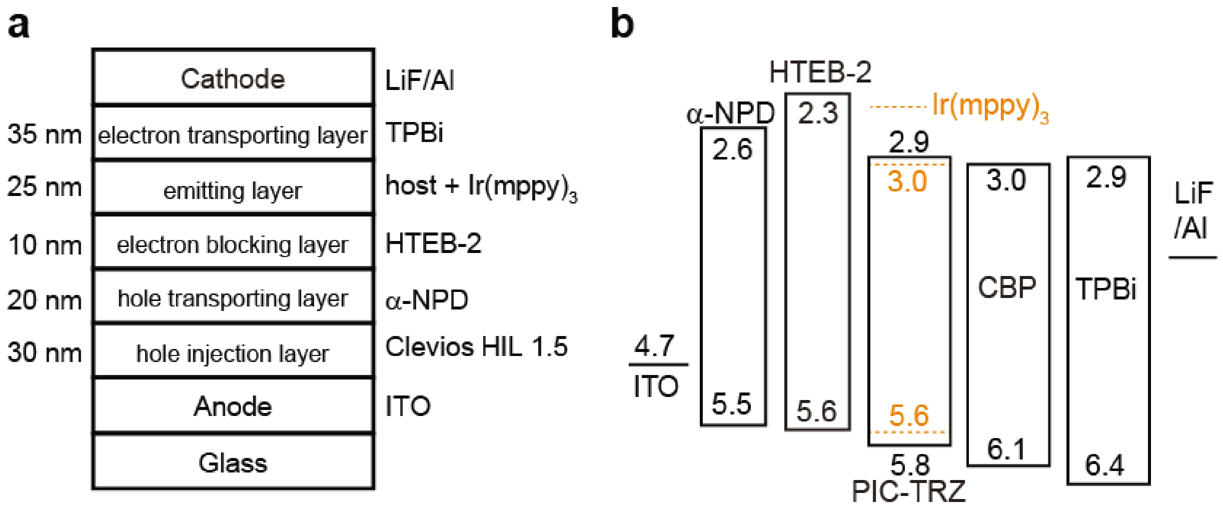

Supplementary Figure S1 | (a) Layer structure of PHOLEDs. (b) Energy level diagram of PHOLEDs. The HOMO level was estimated from the spectroscopic measurement of photoemission in air (AC-3, Rikenkeiki). The LUMO level was estimated by subtracting the optical band gap from the HOMO level. The optical band gap was estimated from the cutoff wavelength of the absorption peak.

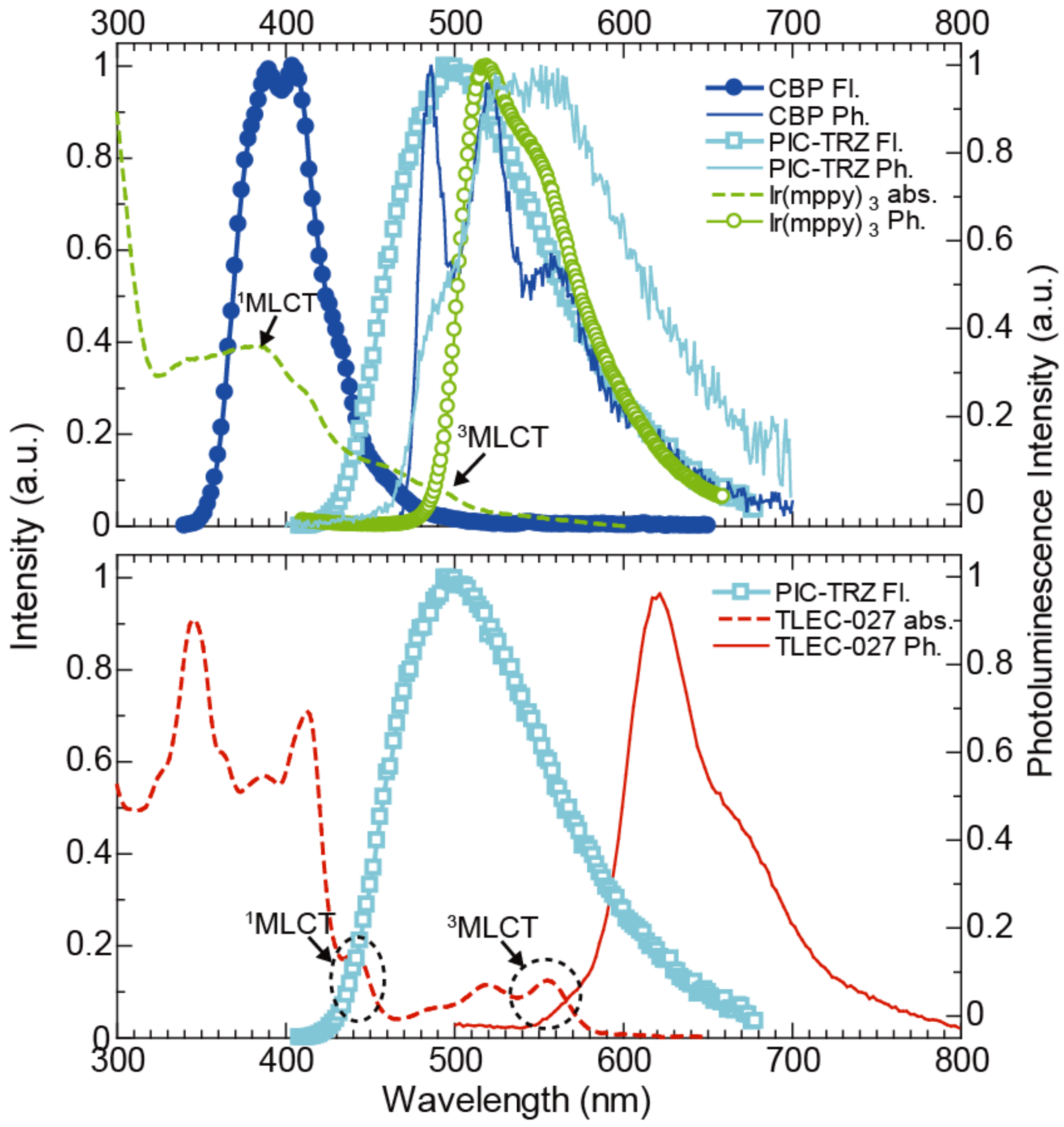

Supplementary Figure S2 | Absorption and emission spectra of the materials. The absorption spectra of Ir(mppy)$_3$ and TLEC-027 were measured at room temperature in dichloromethane (10^{-5} M). The two absorption spectral features appearing on the lower-energy side are likely to arise from the metal-to-ligand charge-transfer transitions (^{1}MLCT and ^{3}MLCT). The photoluminescence (PL) spectra of Ir(mppy)$_3$ and a thin film of CBP doped with TLEC-027 (6 wt%) at room temperature, the fluorescence (Fl.) spectra of the host film measured at room temperature, and the phosphorescence (Ph.) spectra of the host film measured at 10 K are also shown.

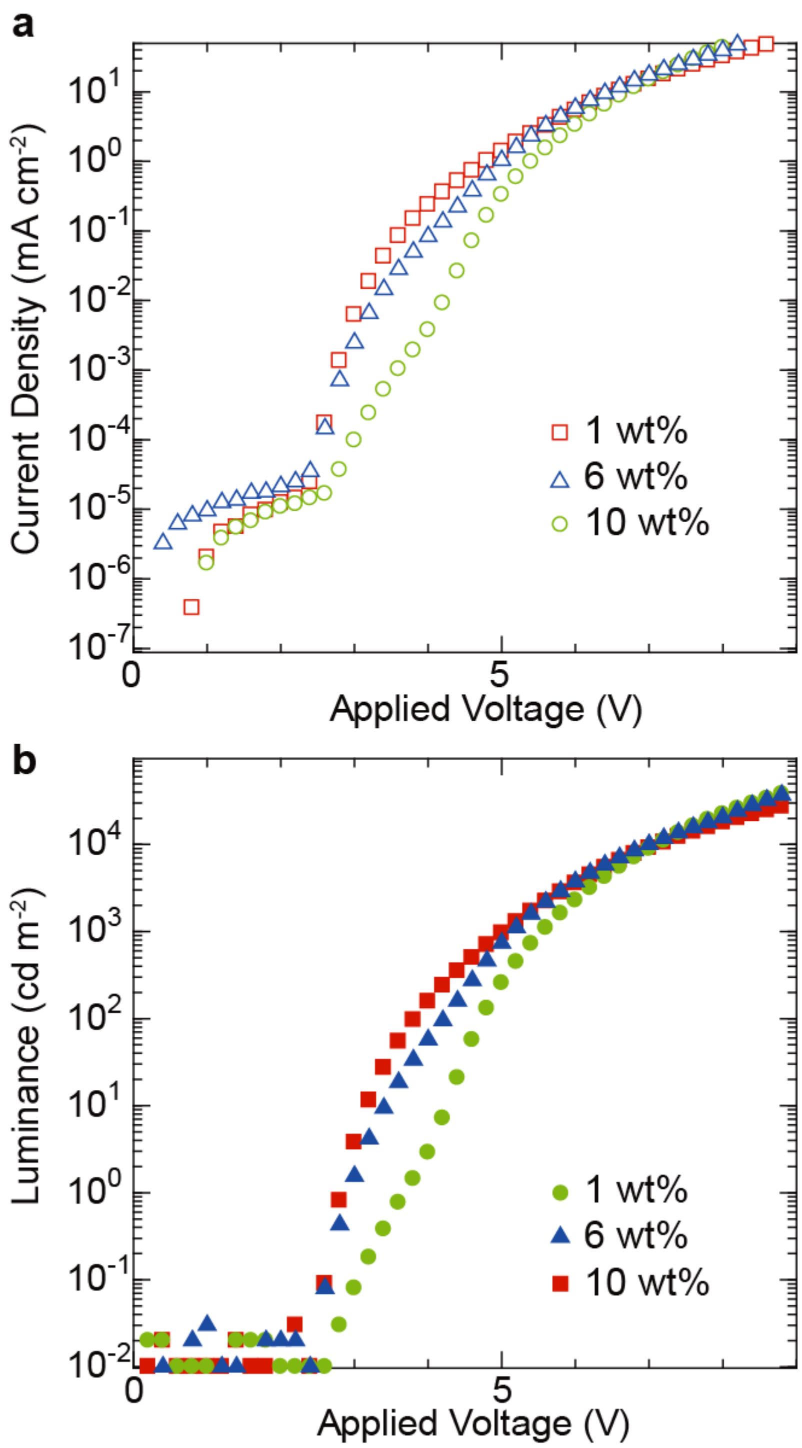

Supplementary Figure S3 | Dopant-concentration-dependent PHOLED characteristics using CBP as a host. (a) Current density–voltage characteristics of PHOLEDs. **(b)** Luminance–voltage characteristics of PHOLEDs.

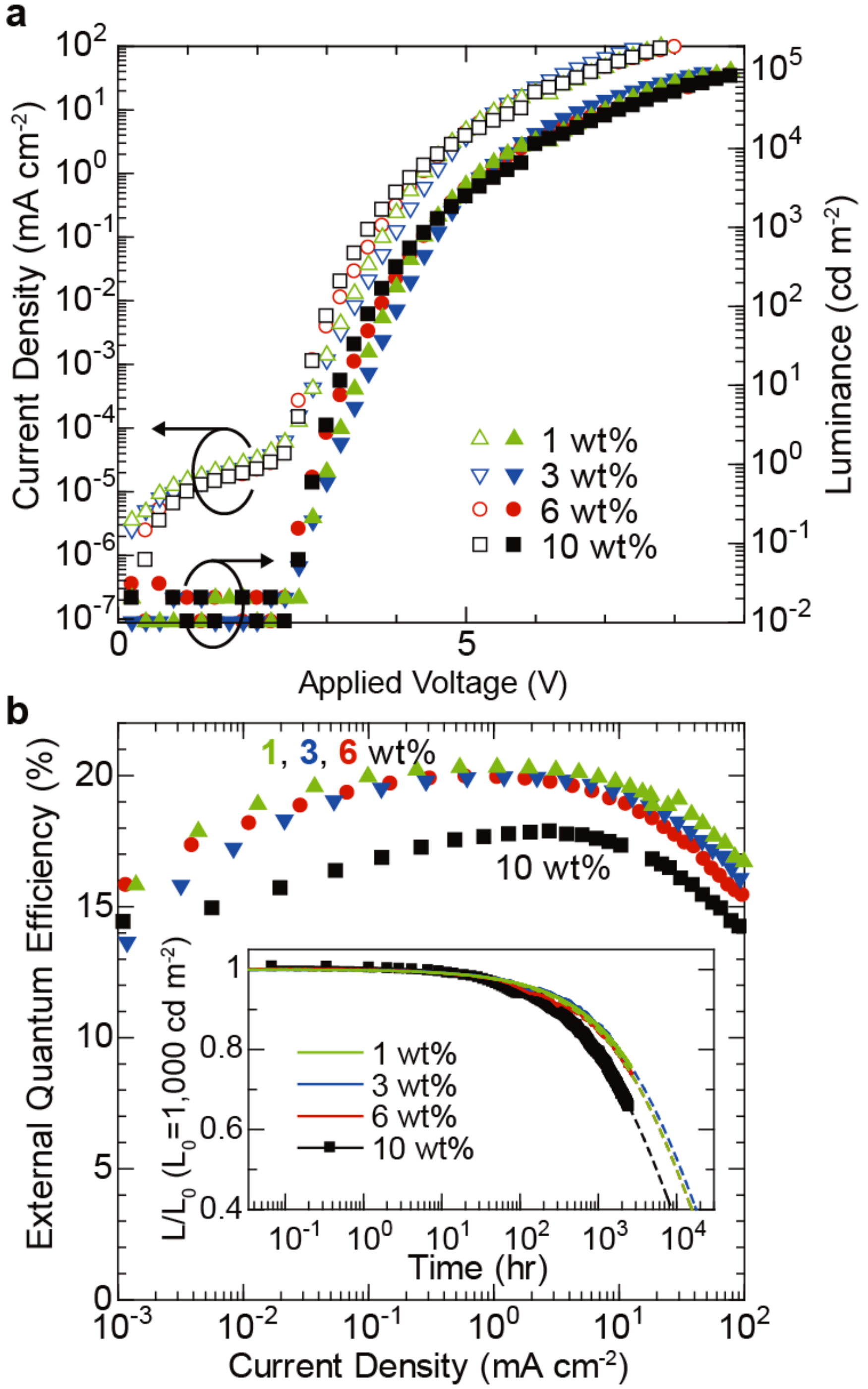

Supplementary Figure S4 | Dopant-concentration-dependent PHOLED characteristics using PIC-TRZ as a host. (a) Current density (left, open symbols) and luminance (right, filled symbols)–voltage characteristics of PHOLEDs. **(b)** External quantum efficiency–current density curves of PHOLEDs. Inset: Luminance–time characteristics for devices under a constant dc current with an initial luminance of 1,000 cd m^{-2}.

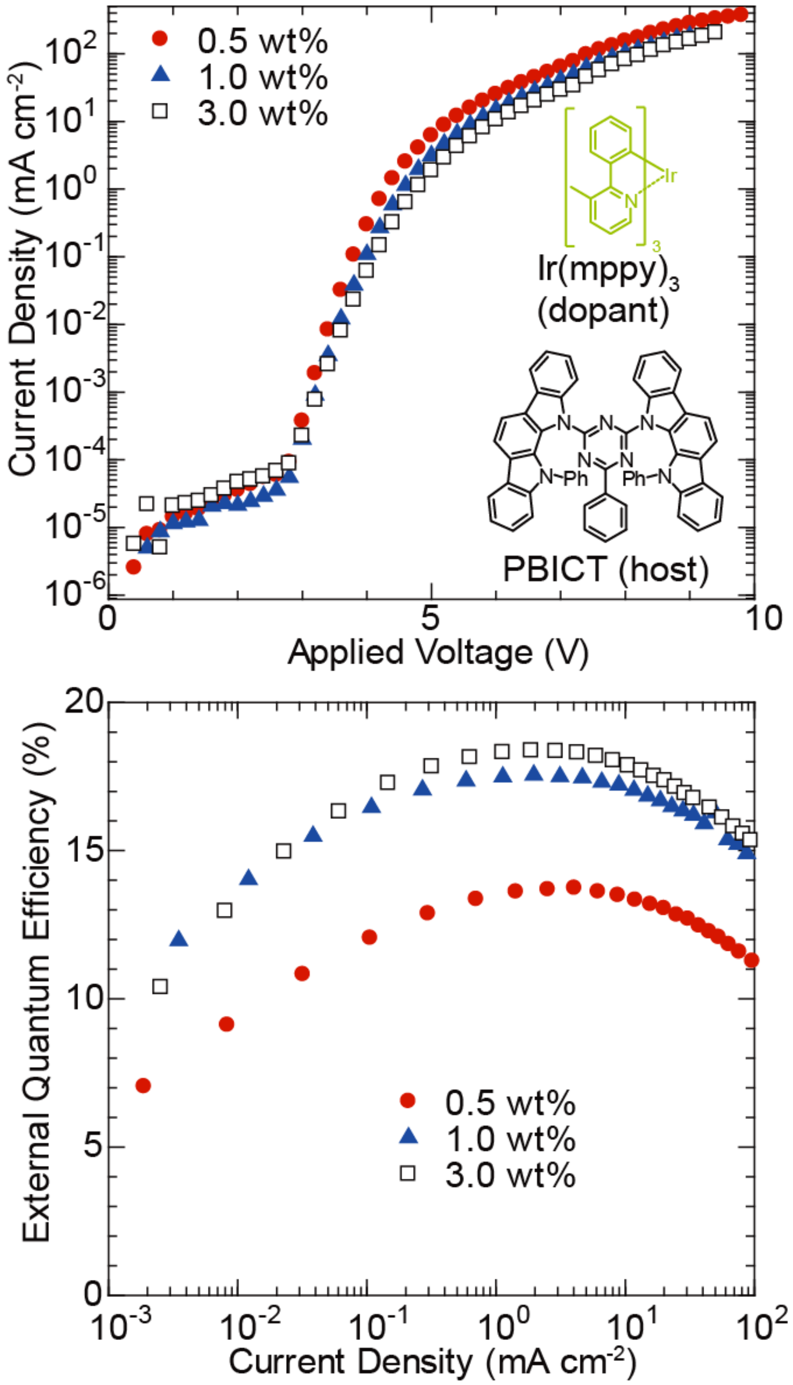

Supplementary Figure S5 | Dopant-concentration-dependent PHOLED characteristics using PBICT as a host. (a) Current density–voltage characteristics of PHOLEDs. **(b)** External quantum efficiency–current density curves of PHOLEDs. The OLEDs were composed of multiple layers of ITO, Clevios HIL 1.5 (30 nm), α-NPD (15 nm), HTEB-2 (10 nm), x wt% Ir(mppy)$_3$:PBICT (20 nm), TPBi (35 nm), LiF (0.5 nm), and Al (100 nm).

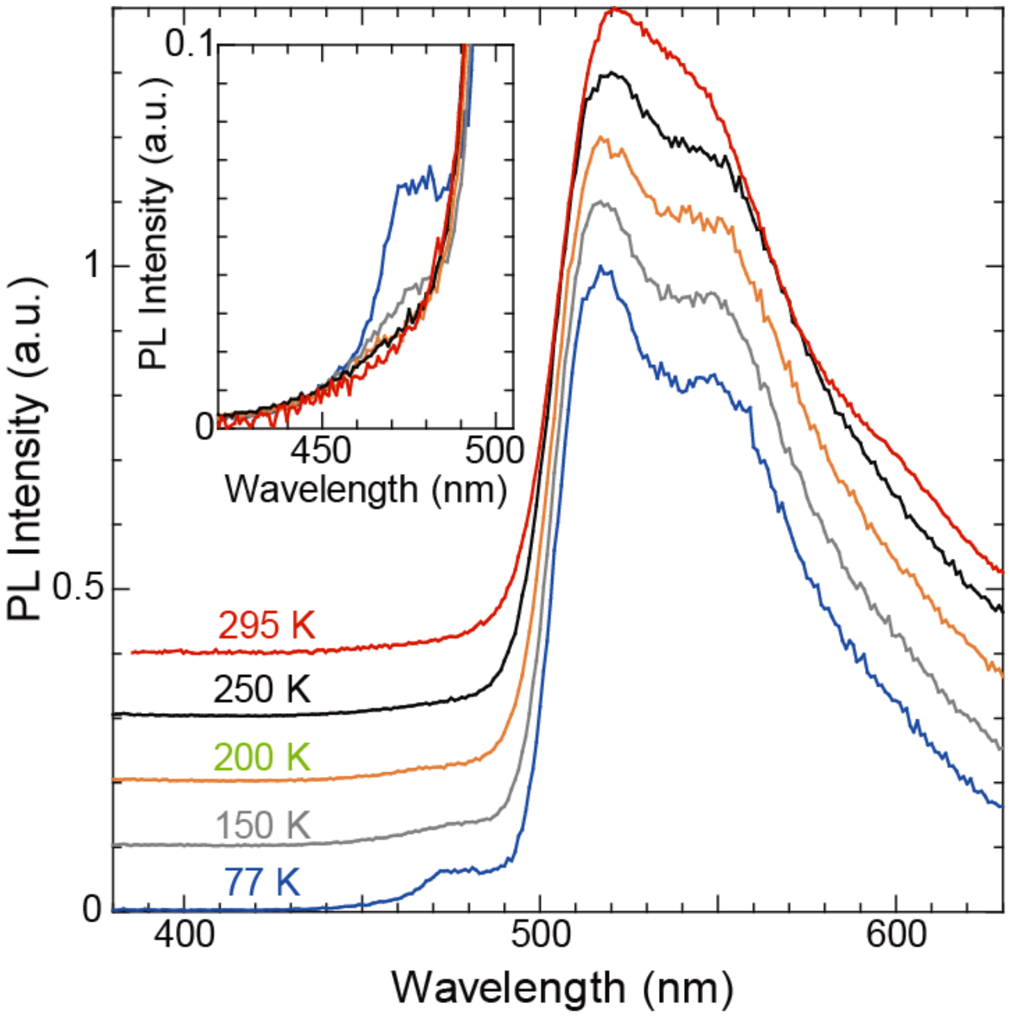

Supplementary Figure S6 | Temperature dependent PL spectrum of three-in-one film, which has the structure 6 wt%-doped FIrpic: 1 wt%-doped Ir(mppy)3: PIC-TRZ film.

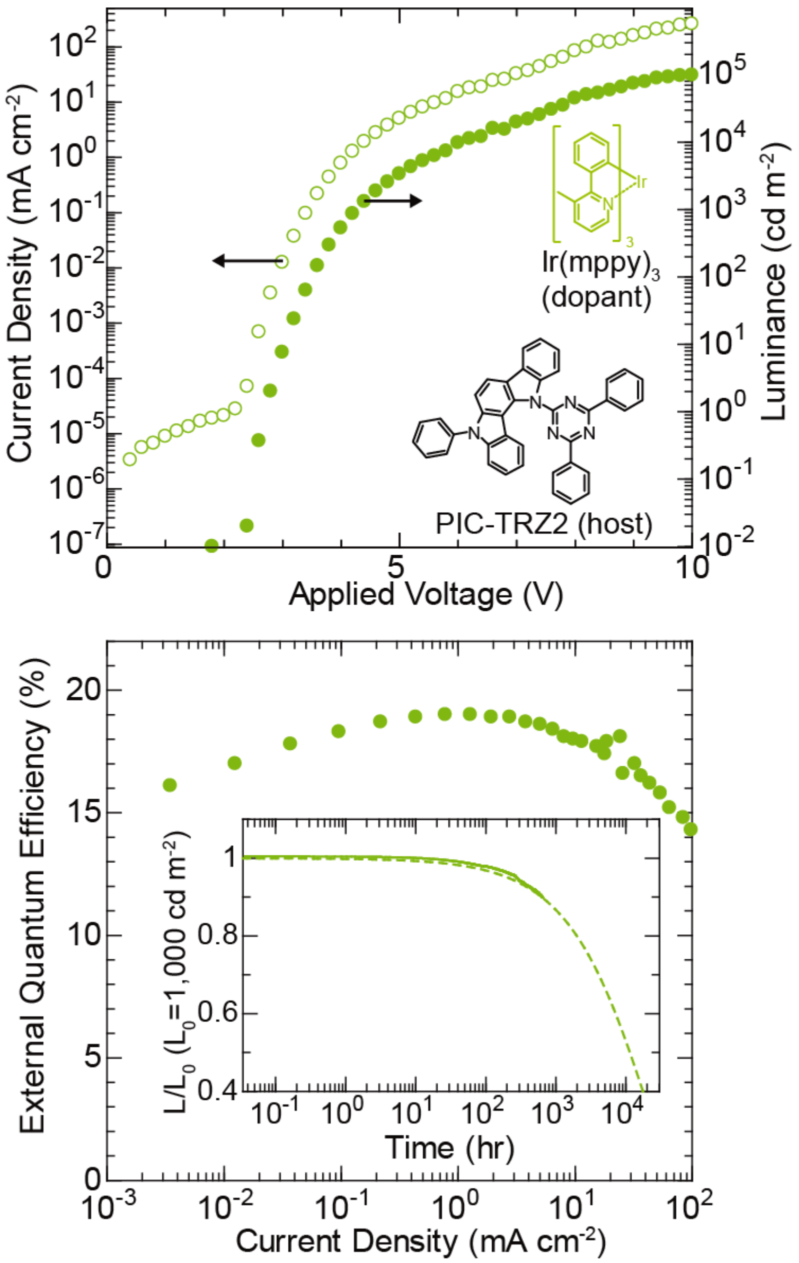

Supplementary Figure S7 | PHOLED characteristics using PIC-TRZ2 as a host. (a) Current density (left, open symbols) and luminance (right, filled symbols)–voltage characteristics of PHOLED. **(b)** External quantum efficiency–current density curves of PHOLED. Inset: Luminance–time characteristics for devices under a constant dc current with an initial luminance of 1,000 cd m^{-2}. The OLEDs were composed of multiple layers of ITO, Clevios HIL 1.5 (30 nm), α-NPD (20 nm), HTEB-2 (10 nm), 1 wt% $Ir(mppy)_3$:PIC-TRZ2 (25 nm), TPBi (35 nm), LiF (0.8 nm), and Al (100 nm).
